# A Retrospective Cohort Study on Rehospitalization following Expanded Criteria Donor Kidney Transplantation

**DOI:** 10.1155/2018/4879850

**Published:** 2018-12-25

**Authors:** Colin Dunn, Emmanuel U. Emeasoba, Michael Hung, Ari Holtzman, Eran Bellin, Stuart Greenstein

**Affiliations:** ^1^Department of Surgery, Albert Einstein College of Medicine, Bronx 10461, NY, USA; ^2^The Montefiore Einstein Center for Transplantation, Montefiore Medical Center, 111 East 210th Street, Bronx 10467, NY, USA

## Abstract

**Background:**

Expanded criteria donor (ECD) kidneys are commonly used but are associated with increased graft failure. Graft failure is in turn related to rehospitalization within thirty days post transplant. Our goal was to determine whether ECD kidneys independently lead to rehospitalization within 30 days, 1 year, and 2 years after transplant.

**Methods:**

All adult first-time recipients of deceased donor kidneys transplanted from 2003–2012 at our center were reviewed. Models included demographics, medical comorbidities, center for disease control high-risk kidney, ECD kidney, ischemia times, cause of renal failure, immunosuppressive regimen, positive psychiatric screening, alcoholism, surgeon, year the transplant was performed, years on dialysis before transplant, and the number of inpatient hospitalizations within 6 months prior to transplant. We conducted Andersen–Gill modeling and propensity score matching followed by logistic regression. We also used multivariable linear regression to predict average length of stay during rehospitalization.

**Results:**

More ECD patients had a rehospitalization at 1 year (70.3% versus 59%, log-rank test *p*=0.014). Thirty-day and 2-year time marks were not significant. Andersen–Gill models predicting successive hospitalizations yielded HR of 1.42 (*p*=0.002) and 1.32 (*p*=0.015) for ECD patients at 1 and 2 years of after transplantation, respectively. Propensity score matching and logistic regression showed a significant relative risk of 1.630 at one year (*p*=0.033) and 1.313 at two years (*p*=0.268). There was no significant association between ECD and subsequent lengths of hospital stay.

**Conclusion:**

Receiving an ECD kidney is independently associated with multiple readmissions within 2 years of transplant but unrelated to length of stay.

## 1. Introduction

Driven by the increasing waiting list and coupled with a diminution in deceased organ donation, kidneys previously considered unacceptable for transplantation have come into widespread use under the sobriquet of “Expanded criteria donor” (ECD) [1]. Recipients of an ECD kidney by definition have a 70% increased risk in graft failure compared to those receiving kidneys from a donor who died from a motor vehicle accident at age 35 [2]. As of 2012, the delayed graft function in the first 90 days after transplant was 30% for ECD kidneys and 20% for standard criteria donors (SCDs), although ninety-day death-censored graft failure was within 2 percentage points [3]. There is accordingly a 30% higher discard rate of ECD kidneys than that of non-ECD kidneys [3]. Additionally, recent publications highlighted an association between receiving an ECD kidney and increased LOS during the initial transplant hospitalization [4–6]. In another paper, patients with extended stays immediately following their transplantation were more likely to have an early readmission [7]. Early readmission to the hospital has proven to be increasingly important because it has been associated with graft loss, mortality, and late hospital readmissions regardless of whether the kidney came from a living or a deceased donor [8]. We hypothesized that the quality of the received graft would affect rehospitalization rates and patient morbidity along with graft survival. We set out to determine whether receipt of an ECD kidney is associated with increased rehospitalization rates at 30 days, 1 year, and 2 years following transplant.

## 2. Materials and Methods

### 2.1. Study Population

After institutional review board approval was obtained from Albert Einstein College of Medicine (IRB number 2014–3276), we utilized the transplant database at the Montefiore Medical Center to identify all patients who had received a deceased donor kidney transplant from January 1, 2003, through December 31, 2012. Pediatric recipients were excluded from the study because they do not receive ECD kidneys at our center. Repeated transplants and multiorgan recipients were also excluded from the study because we did not want prior transplants or other organs to alter the risk for rehospitalization via immune sensitization. HLA mismatch is not a determinant in our institution for determining organ acceptance, and therefore, these data were not analyzed. Patient data were obtained through Clinical Looking Glass (a software/database combination for Montefiore Health System), the Montefiore Transplant Database, and direct review of the electronic medical record [9]. Death was either determined via in-house medical records or Social Security Death Index. ECD kidneys were defined according to the standard United Network for Organ Sharing definition [1].

### 2.2. Definitions

Rehospitalization is defined as any inpatient admission occurring after discharge from kidney transplantation. The Charlson Comorbidity Index is the summation of the patient's medical comorbidities prior to transplant aggregated into one numeric value. The value increases with increasing age from 40 and also increases with the number of medical comorbidities patients had prior to surgery. For further descriptions of weights of different comorbidities, refer [10, 11]. The cause of ESRD was grouped into three categories, each category containing patients who tend to have a similar incidence of disease recurrence after transplant [12]. The first group consisted of focal segmental glomerulosclerosis and IgA nephropathy. The second group consisted of membranous nephropathy, membranoproliferative glomerulonephritis, and lupus nephropathy. The third group consisted of any other cause. To test the assumption of proportional hazards, we performed chi-squared goodness of fit testing between time and each covariate.

### 2.3. Statistical Analysis

The occurrence of a readmission at one and two years after kidney transplant (KT) was demonstrated by cumulative incidence curves (inverse of a Kaplan–Meier). We used the log-rank test at 30 days, 1 year, and 2 years to determine whether there was a significant difference in readmissions at those time points. We used a Bonferroni correction for multiple tests within the same survival plot, with an adjusted alpha of 0.05/3 = 0.0167. Multivariable analysis of readmission was modeled using an Andersen–Gill (AG) analysis, which allowed us to model the proportional effect of covariates on multiple readmissions, not just the hazard ratio for one readmission [13]. For AG, we initially entered all variables that have been shown in the literature to affect graft survival or rehospitalization after a surgical procedure [14–16].

The following variables were considered as potential covariates in AG: age of recipient (dichotomized using an age threshold of 65), race, diabetes, peripheral vascular disease, BMI, center for disease control high-risk kidney, Charlson Comorbidity Index, cold ischemia time, warm ischemia time, ECD kidney, causes of end stage renal disease (ESRD), hepatitis C virus status, induction type (basiliximab or thymoglobulin), immunosuppressive regimen (mycophenolate mofetil (MMF), tacrolimus, and sirolimus), surgeon who performed the transplant, years on dialysis before transplant, year the transplant was performed, and the number of inpatient hospitalizations 6 months before transplant.

For further verification of the AG results, nearest neighbor propensity score matching without replacement or discard was carried out. The ratio of SCDs to ECDs for matching was 3 : 1. The following covariates were used a priori for matching: years on dialysis before transplant, age and cause of ESRD as outlined above, BMI, year transplant was performed, the number of inpatient hospitalizations within 6 months of transplant, race, and Charlson Comorbidity Index. After matching, the resulting weights were used in logistic regression models to predict rehospitalization at 1 and 2 years after transplant.

To examine which covariates were most predictive of mean length of stay (LOS) for all subsequent hospitalizations in the following two years, we used the Leaps algorithm. This algorithm uses an exhaustive search function to find the best covariates to include in a model according to the Bayesian information criterion [17]. For modeling, we limited our patient cohort to patients who were rehospitalized at least once and removed 5 outlier patients who had mean hospitalization times greater than 60 days.

All of the covariates from the AG models were considered potential covariates for LOS model inclusion, except for whether or not the patient was rehospitalized. The ECD status was “forced-into” the algorithm, meaning that all results would include the ECD status.

We considered a *p* value of 0.05 or less statistically significant unless otherwise stated. All confidence intervals are 95%, and all tests are two-tailed unless otherwise noted. Categorical variables were described by absolute numbers and percentages. Continuous variables were described using the mean and standard deviation, or the median if the data were skewed. We employed use of the Mann–Whitney *U* test for some univariate analyses when our data did not appear to fit a normal distribution. All analyses were performed using *R*, an open source statistical computing software, along with associated packages [18]. Tables were created using the “tableone” package [19]. The survival analysis was conducted with the survival and survMisc packages [20, 21]. Propensity score matching used the MatchIt package [22]. The Leaps algorithm was run using the “leaps” package [23].

## 3. Results

### 3.1. Study Population

**Figure 1:**
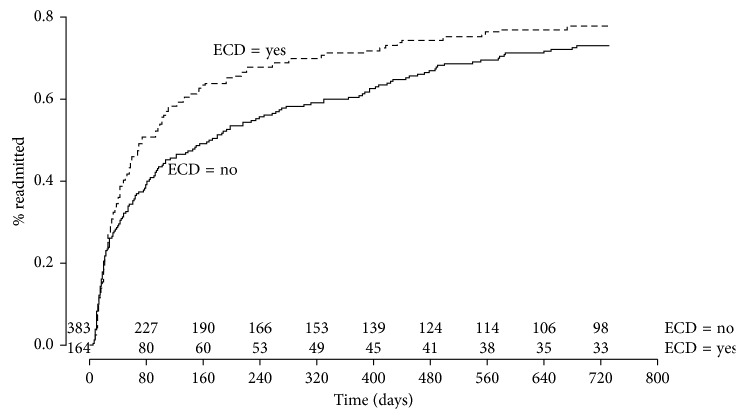
Fraction of patients hospitalized over the course of two years after transplant, stratified by whether the patient received an ECD kidney. Time is in days. The table along the *X*-axis shows raw counts of patients at risk for hospitalization at each time point.

From January 1, 2003, to December 31, 2012, 746 kidney transplants were performed at our institution. After excluding pediatric recipients (79 patients), multiple organ recipients (2 patients), and patients previously transplanted (118 patients), 547 patients were included in our study. In our cohort, the median recipient age was 54 years; 164 (30.01%) patients in our cohort received an ECD kidney. Our cohort was 41.0% Black, 36.7% Hispanic, 10.3% White, and 11.6% other. Median time on dialysis was 4.13 years prior to transplant. Median BMI was 26.72. The median cold ischemia and warm ischemia times were 1285.0 minutes and 40.0 minutes, respectively. Median LOS immediately after transplant was 7.00 days. Comparisons of recipient and donor variables among patients who received ECD and SCD kidneys are shown in (Table 1). There were missing data for induction type (2.01%), warm (1.28%) and cold ischemia times (0.914%), and postoperative LOS (0.236%). These missing values appeared to be missing at random. Of the patients studied, a total of 342 (62.52%) patients were rehospitalized at least once during the first year following KT and 399 (72.94%) patients were rehospitalized at least once during the first 2 years after KT (Figure 1).

There were 74 patients with incomplete follow-up during the study leading to an overall loss of 5.30% of potential follow-up days. Of the 74 patients with incomplete follow-ups, 47 were due to graft failure and 27 were due to early death. Of the 74 patients with incomplete follow-ups, there was no difference between ECD and SCD patient groups for both graft failure and patient death. The other patients in our cohort, including the ECD patient group, were readmitted due to various other reasons. Neither the number of patients with incomplete follow-up (chi-squared, *p*=0.877) nor the number of days missed (Mann–Whitney *U* test, *p*=0.599) varied significantly between the ECD and SCD groups. These same tests also failed to reach significance when only studying patient deaths (chi-square *p*=0.2355, Mann–Whitney U *p*=0.640).

### 3.2. Readmissions following Kidney Transplant

Cumulative incidence curves were used to analyze the number of patients rehospitalized after KT. Thirty days after KT, there was no difference in rehospitalization between ECD and SCD recipients (prevalence 28.7% versus 25.8%, log-rank test *p*=0.658). However, 1 year following KT there was a greater prevalence of rehospitalization among patients who received ECD kidneys (70.73% versus 59%, log-rank test *p*=0.014). This *p* value remained significant after Bonferroni correction for multiple tests (adjusted alpha value=0.017). This significant difference was no longer observed 2 years after KT (76.82% versus 71.28%, log-rank test *p*=0.068).

When considering the potential influence of ECD kidneys on multiple readmissions, we used a one-tailed Mann–Whitney *U* test, due to right-skewed data. Our results suggested that, at both one year (*p* < 0.001) and two years (*p*=0.002), ECDs had a greater number of total readmissions. We further analyzed the effect of the ECD status and other covariates on the risk of multiple successive hospitalizations using Andersen–Gill (AG) models (Table 2). AG models constructed for rehospitalization at 1 and 2 years after transplant showed the ECD status as significant with a hazard ratio in 1 year of 1.42 (*p*=0.002) and 2 years of 1.32 (*p*=0.015). Other significant variables in our 1-year AG model were time spent on dialysis prior to transplantation and total number of admissions 6 months before transplantation (Table 3). Time of dialysis was no longer significant at 2 years (*p*=0.065). The AG model we employed has the same required assumptions as a Cox proportional-hazards model, mainly that variables affect the outcome proportionally over time. We tested this assumption of proportionality by graphing the Schoenfeld residuals and also by performing chi-squared goodness of fit tests between each covariate and time, transformed according to the Kaplan–Meier curve. There was a statistically significant departure from proportionality for both ECD (*p*=0.023) and number of admissions 6 months prior to transplant (*p* ≤ 0.001). Therefore, the hazard ratios for ECD and previous admissions were not constant with time. To further validate that the differences in rehospitalization at 1 and 2 years were in fact due to ECD status and not the other variables, and propensity-score matched logistic regression was performed to isolate the effect of the ECD status alone. The relative risk (RR) for rehospitalization was found to be significant for the ECD status at 1 year (RR = 1.630, *p*=0.033) but insignificant at 2 years (RR = 1.313, *p*=0.268).

## 4. Discussion

Our study demonstrated that receipt of an ECD kidney led to a 63% increase in risk for rehospitalization within the first year. All inpatient readmissions occurred exclusively at our center. A statistically significant association was present irrespective of whether an AG model or a propensity score matched logistic regression model was used. We however found a statistically significant departure from the proportional hazards assumption in our AG model. While this does not invalidate our results, it suggests that the effect of those variables is more complex than the assumption of proportionality would allow. Put differently, the strength of the effect these variables have on rehospitalization changes with time. This makes sense because ECDs were significantly associated with rehospitalization at 1 year, but not 30 days or 2 years after transplant according to our log-rank tests.

This study highlights the importance of considering alternative metrics when evaluating the equivalence of ECD kidneys to SCD kidneys. Many studies have compared the two kidney groups on the basis of delayed graft function and overall graft survival. We believe that rehospitalization is a worthy metric in its own right. The various reasons for readmission and/or hospital charges for all readmitted patients are not available. By 2006, rehospitalization after kidney transplant already cost an average of 13,678 dollars [24]. In addition to cost, there is also the quality of life of the patient to consider. Acquired disability through immobility in the hospital is not uncommon among the elderly [25].

Studies have shown that patients over the age of 60 take longer time to recover from surgery than previously believed [26–28]. In a study by Mayo et al. by 6 months, fewer than 50% of patients who underwent major abdominal surgery had recovered their baseline levels of physical performance [28]. This implies that many of the patients in our cohort would be back in the hospital before they could even recover from their surgery. Because we made every effort to adjust for confounding variables in our study, we believe that the rehospitalizations are caused by the quality of the graft itself.

There are few studies which compared ECD versus SCD in relation to rehospitalization. A study combining retrospective and prospective data conducted by Stratta et al. in 2006 found no association between ECDs and rehospitalization with just 77% of patients followed for a year or more [29]. We believe the difference in observational data may have contributed to their lack of statistical significance. Furthermore, this study did not use multivariable regression. Another study published by Harhay et al. also did not find any association between ECD status and rehospitalization [30]. However, they only looked at rehospitalizations within 30 days of discharge. When we examined this time mark, we also failed to find any association between ECD and rehospitalization.

Although the ECD status was significantly associated with rehospitalization, it did not reach statistical significance for predicting mean LOS. Mean LOS was significantly associated with a history of alcoholism, taking sirolimus, or having a longer initial length of stay. The most clinically significant of these would be alcoholism and initial length of stay, as they had larger effect sizes.

A recent study reinforced the risk of using ECD kidneys, finding adjusted hazard ratios of 1.46 to 1.20 for all-cause mortality in different age groups up to age 70 [31]. Other studies have found increased incidences of delayed graft function with ECD kidneys [32]. There have been many studies attempting to mitigate the risk of ECD kidney usage. Studies have argued for decreased cold ischemia time, using perfusion pumps rather than simple cold storage, selective use of ECDs for recipients with low PRA, or refinement of predictors for graft failure through kidney biopsy [33–39]. Some of these studies report promising results using combinations of all of these techniques [40]. More recently, there has been progress diagnosing acute rejection through use of ultrasound or urine metabolites, which may improve the longevity of the allograft [39, 41]. Others have argued even further that ECD kidneys may be selectively used when they are donated after cardiac death (DCD) [42].

This study has important limitations, one of which is the retrospective design. Although every effort was made to include confounding factors into the multivariable analysis, it is possible that there is a confounding variable which influences both ECD/SCD status and rehospitalization rates. Only a randomized controlled trial can fully eliminate unforeseen biases. Another limitation of our study was missing data. Although the vast majority of patients in our study had complete follow-up time and follow-up time did not differ between ECD and SCD recipients, it is possible that those patients lost to follow-up could be vastly different from the patients who completed the study, hence affecting our results. HLA mismatch data were not analyzed for our study, and therefore, recipient immunological risk at transplant might be incompletely controlled in our study.

This study utilized a unique patient population (Bronx, NY). We have a higher proportion of Black and Hispanic patients than other transplant centers [43]. Furthermore, 29.2% of our patients are living below the poverty line [43]. Although we attempted to “control” for demographic factors through multivariable modeling, the ECD effect we observed may differ from other populations.

Importantly, our results use an aging metric to assess kidney quality. The Kidney Donor Profile Index (KDPI) and Kidney Donor Risk Index (KDRI) have recently supplanted the classification of ECD/SCD [44]. Our continued research will evaluate whether the Kidney Donor Risk Index (KDRI) is also associated with readmission. Additionally, newer studies have suggested that kidney biopsy may be a more effective way of predicting graft longevity [45]. There has been an attempt to standardize the biopsy-reading process with specialized pathologists and scoring systems [40,46–48]. These scoring systems may supplement the predictive power of the KDPI. We hypothesize that poor graft quality as evidence by kidney biopsy will also be associated with increased rehospitalizations.

## 5. Conclusion

After extensive multivariable modeling and propensity score matching, we observed an association between receipt of an ECD kidney and increased risk for multiple rehospitalizations within 1–2 years after transplant. This effect should be replicated and further characterized. Patients should fully understand their risks before choosing an ECD kidney.

## Figures and Tables

**Table 1:** Comparing baseline characteristics between SCD and ECD kidney recipients.

*n*		SCD	ECD
		383	16
Admit count 180 days before KT, mean (SD)	0.0.234 (0.625)	0.274 (0.746)	
Age, mean (SD)		50.196 (12.725)	59.049 (9.756)
Total rehosp, median [IQR]		1.000 [0.000, 3.000]	2.000 [1.000, 4.000]
ESRD cause category, *n* (%)			
** **FSGS or IgA		29 (7.6)	9 (5.5)
** **Membranous or MPGN or lupus		31 (8.1)	5 (3.0)
** **Other		323 (84.3)	150 (91.5)
Induction = thymoglobulin, *n* (%)		311 (83.4)	130 (79.8)
MMF, *n* (%)		270 (70.5)	123 (75.0)
Sirolimus, *n* (%)		166 (43.3)	72 (43.9)
Tacrolimus, *n* (%)		373 (97.4)	161 (98.2)
CDC, *n* (%)		61 (15.9)	8 (4.9)
DCD, *n* (%)		70 (18.3)	12 (7.31)
Years of dialysis before KT, mean (SD)	4.747 (4.009)	4.092 (3.429)	
HCV, *n* (%)		21 (5.5)	6 (3.7)
BMI, mean (sd)		27.468 (5.854)	27.248 (5.300)
CIT, mean (sd)		1334.508 (658.539)	1382.390 (601.300)
WIT, mean (sd)		42.024 (12.696)	40.256 (12.979)
Charlson Index, median [IQR]		2.000 [2.000, 4.000]	2.000 [1.000, 4.000]
Length of post-op stay, median [IQR]		7.000 [6.000, 9.000]	7.000 [6.000, 10.250]
Diabetes, *n* (%)	No	223 (58.2)	80 (48.8)
	Yes	160 (41.8)	84 (51.2)
PVD, *n* (%)	No	339 (88.5)	148 (90.2)
	Yes	44 (11.5)	16 (9.8)
Surgeon, *n* (%)	0	2 (0.5)	0 (0.0)
	1	64 (16.7)	29 (17.7)
	2	31 (8.1)	12 (7.3)
	3	45 (11.7)	18 (11.0)
	4	50 (13.1)	33 (20.1)
	5	55 (14.4)	22 (13.4)
	6	75 (19.6)	28 (17.1)
	7	61 (15.9)	22 (13.4)

Format is absolute value (percent within group). ESRD = end-stage renal disease, FSGS = focal segmental glomerulosclerosis, IgA = IgA nephropathy, Mem = membranous nephropathy, MPGN = membranoproliferative glomerulonephritis, Lupus = lupus nephritis, IQR = interquartile range, MMF = mycophenolate mofetil, CDC = Center for Disease Control high-risk kidney, DCD = donated after cardiac death, KT = kidney transplant, HCV = hepatitis C virus, CIT = cold ischemia time, WIT = warm ischemia time, post-op = postoperative, PVD = peripheral vascular disease ^*∗*^Each number in the second column represents a specific surgeon who performed the operation.

**Table 2:** Results of the 1- and 2-year Andersen–Gill (AG) model.

Variable	1 yr HR	1 yr 95% CI	1 yr *p* value	2 yr HR	2 yr 95% CI	2 yr *p* value
ECD	1.43	[1.14,1.79]	^*∗*^0.002	1.32	[1.06, 1.65]	∗0.015
Years on dialysis	1.04	[1, 1.07]	^*∗*^0.03	1.03	[0.998, 1.07]	0.065
Admissions 180 d before transplant	1.18	[1.02,1.38]	^*∗*^0.029	1.22	[1.06, 1.41]	∗0.005
Race black	1.23	[0.912, 1.67]	0.173	1.22	[0.915, 1.63]	0.176
Race hispanic	1.35	[0.993, 1.83]	0.055	1.32	[0.975, 1.79]	0.072
Age >65	1.24	[0.924, 1.66]	0.153	1.22	[0.924, 1.61]	0.16
ESRD, FSGS, or IgA	0.734	[0.491,1.1]	0.131	0.75	[0.527, 1.07]	0.109
ESRD membranous, MPGN, or lupus	0.764	[0.508, 1.15]	0.196	0.752	[0.534, 1.06]	0.104
Basiliximab	0.89	[0.654, 1.21]	0.461	0.909	[0.68, 1.21]	0.515
MMF	1.23	[0.85,1.79]	0.271	1.15	[0.827, 1.59]	0.41
Sirolimus	0.945	[0.715, 1.25]	0.689	0.929	[0.712, 1.21]	0.589
Tacrolimus	2.12	[0.637, 7.02]	0.221	1	[0.56, 1.79]	0.996
CDC	1.02	[0.764, 1.36]	0.896	1.07	[0.796, 1.43]	0.667
HCV	1.24	[0.763, 2.02]	0.384	1.4	[0.865, 2.26]	0.171
BMI	1.01	[0.99,1.02]	0.433	1.01	[0.996, 1.03]	0.139
CIT	1	[1, 1]	0.933	1	[1, 1]	0.985
WIT	1	[0.995, 1.01]	0.398	1	[0.993, 1.01]	0.721
Charlson Index	1.03	[0.981, 1.08]	0.221	1.04	[0.991, 1.08]	0.114
Length of stay	1.01	[1, 1.01]	0.063	1.01	[0.999, 1.01]	0.092
Surgeon 1	1.13	[0.739, 1.72]	0.580	1.11	[0.748, 1.65]	0.599
Surgeon 2	1.2	[0.765, 1.87]	0.432	1.16	[0.745, 1.8]	0.514
Surgeon 3	0.915	[0.595, 1.41]	0.687	0.883	[0.559, 1.4]	0.595
Surgeon 4	1.2	[0.807, 1.77]	0.372	1.24	[0.856, 1.81]	0.254
Surgeon 5	0.906	[0.554, 1.48]	0.695	0.879	[0.558, 1.38]	0.577
Surgeon 6	1.23	[0.691, 2.2]	0.479	1.16	[0.584, 1.97]	0.584

^*∗*^ *p* values<0.05. HR = hazard ratio; CI = confidence interval; ECD = expanded criteria donor kidney; CIT = cold ischemia time; WIT = warm ischemia time.

**Table 3:** Results of the logistic regression following propensity score matching.

Variable	1 year RR	2.50%	97.50%	*p* value	2 year RR	2.50%	97.50%	*p* value
ECD	1.63	1.04	2.572	^*∗*^0.033	1.31	0.81	2.14	0.26
Age at transplant	1.01	0.99	1.029	0.15	1.00	0.99	1.02	0.36
SES	0.96	0.90	1.036	0.35	0.97	0.90	1.04	0.45
Simulect	0.76	0.44	1.300	0.31	0.74	0.42	1.32	0.30
MMF	1.92	1.11	3.339	^*∗*^0.019	1.74	0.96	3.19	0.06
Sirolimus	1.09	0.66	1.847	0.71	1.31	0.75	2.34	0.34
Tacrolimus	3.59	0.92	17.970	0.08	1.85	0.48	7.02	0.35
CDC	1.10	0.62	1.977	0.74	1.36	0.73	2.68	0.34
Years of dialysis	1.04	0.98	1.100	0.15	1.01	0.96	1.07	0.56
HCV	1.35	0.54	3.697	0.53	1.27	0.47	3.95	0.64
BMI	1.00	0.96	1.037	0.88	1.01	0.97	1.04	0.57
Charlson Index	1.08	0.99	1.192	0.08	1.07	0.97	1.19	0.14

^*∗*^ *p* values <0.05. RR = relative risk; CI = confidence interval; ECD = expanded criteria donor kidney; SES = socioeconomic status; MMF = mycophenolate mofetil; CDC = Center for Disease Control high-risk kidney; HCV = hepatitis C virus; BMI = body mass index.

## Data Availability

The data used to support the findings of this study have not been made available because of patient confidentiality.

## Abbreviations

ECD: Expanded criteria donor
SCD: Standard criteria donor
DCD: Donated after cardiac death
CDC: Center for Disease Control
WIT: Warm ischemia time
CIT: Cold ischemia time
KT: Kidney transplant
HCV: Hepatitis C virus
LOS: Length of stay.
